# Supplementary material for: Wetting Properties of Graphene Aerogels
Source: Sci Rep. 2020 Feb 5;10:1916. doi: 10.1038/s41598-020-58860-4 (PMC7002654; doi:10.1038/s41598-020-58860-4)
Supplement: Supplementary file 2 — Supplementary Information2. [file 41598_2020_58860_MOESM2_ESM.pdf]

Supplementary Information to Wetting Properties of Graphene Aerogels

Francesco De Nicola,[1,*] Ilenia Viola,[2] Lorenzo Donato Tenuzzo,[3] Florian Rasch,[4] Martin R. Lohe,[5] Ali Shaygan Nia,[5] Fabian Schütt,[4] Xinliang Feng,[5] Rainer Adelung,[4] and Stefano Lupi[1,3]

[1]*Graphene Labs, Istituto Italiano di Tecnologia, Via Morego 30, 16163 Genova, Italy*

[2]*CNR NANOTEC-Institute of Nanotechnology, S.Li.M. Lab, Department of Physics, University of Rome La Sapienza, P.le A. Moro 5, 00185 Roma, Italy*

[3]*Department of Physics, University of Rome La Sapienza, P.le A. Moro 5, 00185 Roma, Italy*

[4]*Functional Nanomaterials, Institute for Materials Science, Kiel University, Kaiser Str. 2, 24143 Kiel, Germany*

[5]*Center for Advancing Electronics Dresden (CFAED) & Department of Chemistry and Food Chemistry, Technische Universität Dresden, Helmholtzstraße 10, 01069 Dresden, Germany*

RAMAN CHARACTERIZATION

Raman spectra were acquired by a Jasco NRS-5100 with a 532 nm laser source and a 100× microscope objective. In Figure 1a, a representative Raman spectrum of a ZnO aerogel template is reported. The Zn and O bands[1] at 439 cm^3 and 1153 cm^3, respectively, can be observed. In Figure 1b, a representative Raman spectrum of an Aerographene aerogel sample after the chemical etching to remove the ZnO template is shown. The typical graphene features can be observed. The D, G, and 2D bands are at 1347 cm^3, 1575 cm^3, and 2680 cm^3, respectively. The Zn and O bands cannot be observed anymore.

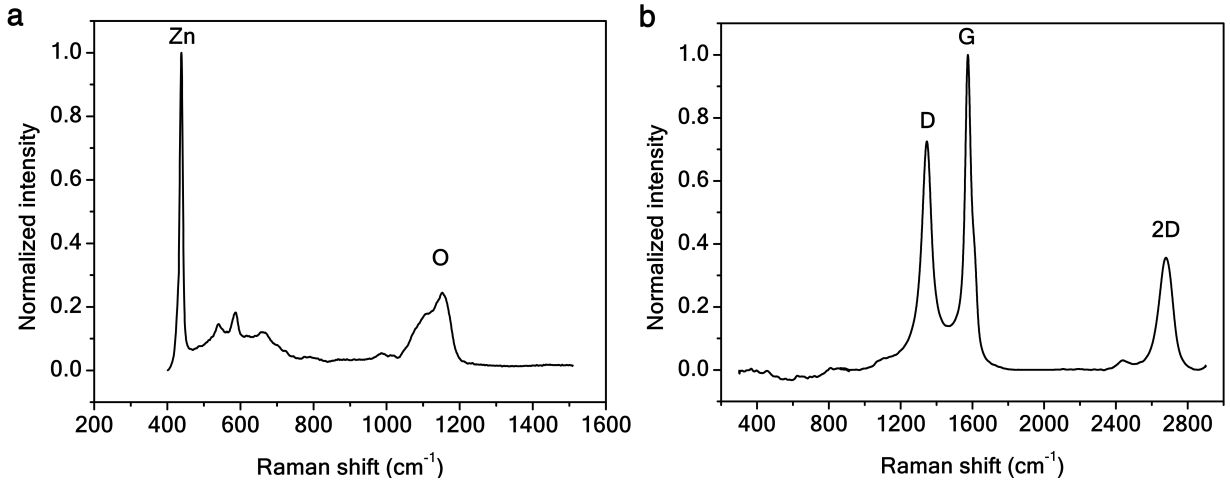

FIG. 1. Representative Raman spectra of a ZnO aerogel template (**a**) and an Aerographene aerogel after the chemical etching (**b**).

* E-mail: francesco.denicola@iit.it

1 Calleja, J. M. & Cardona, M. Resonant raman scattering in zno. *Phys. Rev. B* **16**, 3753–3761 (1977).
